# Illegal tusk harvest and the decline of tusk size in the African elephant

**DOI:** 10.1002/ece3.1769

**Published:** 2015-10-22

**Authors:** Patrick I. Chiyo, Vincent Obanda, David K. Korir

**Affiliations:** ^1^ Department of Biology Duke University Box 90338 Durham North Carolina 27708; ^2^ Veterinary Services Department Kenya Wildlife Service P.O. Box 40241‐00100 Nairobi Kenya; ^3^ Biodiversity Monitoring & Research Division Kenya Wildlife Service Masai Mara Research Station P.O. Box 72‐20500 Narok Kenya

**Keywords:** Anthropogenic impacts, evolution of morphology, hunting, inheritance of incisors, ivory, selection, tusk evolution, tusklessness

## Abstract

Harvesting of wild populations can cause the evolution of morphological, behavioral, and life history traits that may compromise natural or sexual selection. Despite the vulnerability of large mammals to rapid population decline from harvesting, the evolutionary effects of harvesting on mega‐fauna have received limited attention. In elephants, illegal ivory harvesting disproportionately affects older age classes and males because they carry large tusks, but its' effects on tusk size for age or tusk size for stature are less understood. We tested whether severe historical elephant harvests eliminated large tuskers among survivors and whether elephants born thereafter had smaller tusks. Adjusting for the influence of shoulder height – a metric strongly correlated with body size and age and often used as a proxy for age – we compared tusk size for elephants sampled in 1966–1968, prior to severe ivory harvesting in the late 1970s and early 1980s, with tusk size of survivors and elephants born during population recovery in the mid‐1990s. In a regional population, tusk length declined by ˜21% in male and by ˜27% in female elephants born during population recovery, while tusk length declined by 22% in males and 37% in females among survivors. Tusk circumference at lip declined by 5% in males but not in females born during population recovery, whereas tusk circumference reduced by 8% in male and by 11% in female survivors. In a single subpopulation, mean tusk length at mean basal tusk circumference declined by 12.4% in males and 21% in females. Tusk size varied between elephant social groups. Tusk homogeneity within social groups and the often high genetic similarity within social groups suggest that tusk size may be heritable. Our findings support a hypothesis of selection of large tuskers by poachers as a driver of the decline in tusk size for age proxy and contemporary tusk evolution in African elephants.

## Introduction

Human‐induced environmental changes and harvesting of wild populations are increasingly recognized as important agents of contemporary evolution, sometimes outpacing natural agents as drivers of phenotypic change (Hendry et al. 2008; Darimont et al. 2009; Pelletier et al. 2012). Harvesting of vertebrate populations can cause the evolution of morphological, life history, and behavioral traits that may be antagonistic to natural or sexual selection (Law 2001; Festa‐Bianchet 2003; Carlson et al. 2007; Hengeveld and Festa‐Bianchet 2011; Ciuti et al. 2012). As natural selection brings about local adaptation to the natural environment, selective harvesting can impede adaptive evolutionary processes, exacerbate extinction risk from stochastic demographic processes, and may delay the recovery of populations following reduction or elimination of harvesting (Olsen et al. 2004; Allendorf and Hard 2009; Edeline et al. 2009).

Selective harvesting and severe harvesting are expected to produce similar demographic and evolutionary effects on life history traits. Vertebrate populations experiencing sustained size‐selective harvesting often show a shift in age structure leading to a predominance of young age classes and a reduction in older age classes because the traits targeted by hunters are usually positively correlated with age. A change in age structure is usually accompanied by reduction in trait size for age particularly when some variation in trait size for age within age cohorts exists (Coltman et al. 2003; Garel et al. 2007; Festa‐Bianchet et al. 2014). Selective harvesting focused on sexually dimorphic traits can lead to a change in population sex ratio and a reduction in size of the selected trait for age (Garel et al. 2006; Milner et al. 2007). This is because changes in age and sex structure in polygynous species resulting from selective harvesting are known to relax sexual selection pressures and to favor the development of small body size for age and small horn size for age in many ungulates (Mysterud et al. 2005; Tiilikainen et al. 2010). Aside from selective harvesting, there is also theoretical and empirical evidence showing that increased mortality due to severe nonselective harvesting can lead to reduction in body size for age as a result of selection for faster maturation schedules (Olsen et al. 2004, 2005; Engen et al. 2014). Specifically, high mortality selects for early maturation at a small size because under a higher harvest pressure, individuals that begin reproduction at younger ages and at small body sizes have a greater chance of reproducing compared with individuals that become reproductively mature at older ages and large body sizes (Proaktor et al. 2007). Empirical support for this hypothesis comes mostly from fish (Law 2000, 2001; Olsen et al. 2005; Reznick and Ghalambor 2005; Carlson et al. 2007; Swain et al. 2007; Diaz Pauli and Heino 2014; Kendall et al. 2014), but few studies have tested this proposition in mammals (Mysterud et al. 2009; Prowse et al. 2015).

The effects of selective or severe harvesting on the evolution of morphological and life history traits in wild vertebrate populations are well studied in fish and a few large mammals (Fenberg and Roy 2008). There is a dearth of studies focusing on the effects of selective or severe harvesting on phenotypic change in mega‐herbivores: herbivores with an adult body mass equal to or greater than 1000 kg (Owen‐Smith 1992). In addition, studies on phenotypic and evolutionary effects of selective or severe harvesting have focused mostly on temperate vertebrates leaving scarcity of data regarding the selective effects of illegal harvesting in the tropics where such harvests are rampant. Elephants are such one species with a history of illegal harvesting (Spinage 1973) for which phenotypic effects of illegal hunting have received less attention (Jachmann et al. 1995).

Efforts to determine the consequences of illegal harvesting on African elephants have focused on its effects on their population sizes and life history (Barnes and Kapela 1991; Prins et al. 1994; Gobush et al. 2008; Bouche et al. 2011). Illegal ivory harvesting caused unprecedented decline in elephant populations across the continent in the late 1970s and early 1980s; some protected areas in East Africa lost as much as 50–90% of their elephant numbers (Eltringham and Malpas 1980; Douglas‐Hamilton 1987; Ottichilo et al. 1987; Barnes and Kapela 1991; Prins et al. 1994). Illegal ivory harvesting caused changes in age and sex structure leading to reduction or elimination of older animals; an effect more severe in males compared to females (e.g., Poole 1989; Poole and Thomsen 1989; Barnes and Kapela 1991). Elephant poaching targets older individuals and males because older animals and males carry large tusks than younger animals and males carry larger tusks compared to females. The preference for older age classes and the male sex by poachers suggests selective harvesting of large tuskers because such animals provide a higher monetary return for each animal killed. Moreover, the potential for selective harvesting by poachers is likely to be high for savannah elephants because they live in large groups and in open savannah landscapes where visibility can favor the detection and selection of animals carrying large ivory. Such selective harvest can decrease the survival of elephants with large tusks for age relative to the survival of animals with smaller tusks for age. Although selective harvests of large‐tusked elephants for body size by poachers is possible, it has not been empirically tested. Most studies on the phenotypic effects of ivory harvesting on elephants have focused on tusklessness (Jachmann et al. 1995; Kurt et al. 1995; Abe 1996; Whitehouse 2002) probably because it is easily detectable and does not require any measurements. These studies have however shown that the incidence of tusklessness increases in local elephant populations subject to heavy illegal harvesting (Jachmann et al. 1995; Whitehouse 2002).

In this study, we tested the effects of illegal harvesting on changes in tusk size by comparing tusks from elephants captured in southern Kenya between 2005 and 2013 with tusks of elephants culled between 1966 and 1968 from the same region. Comparative analysis of morphological traits of survivors with traits of the original populations to provide evidence of selection in different vertebrate species has a rich history (e.g., Bumpus 1899; Endler 1986; Shine et al. 2001; Garel et al. 2007; Festa‐Bianchet et al. 2014). We take advantage of this approach to detect selection for large tusks by illegal harvesting in our analyses. We specifically tested the hypotheses that: (1) contemporary elephants born before or by 1970 have small tusks for stature or body size as a result of the elimination of animals with large tusks for stature or body size; (2) contemporary elephants born in 1995 onwards or offspring of survivors of the 1970s and 1980s illegal ivory harvest have small tusks for age or stature compared to individuals of similar age or stature from the 1966–1968. The mid‐1990s was a period of increasing elephant populations recovering from illegal killing of elephants for ivory in East Africa (Litoroh 2003; Blanc et al. 2005; Wittemyer et al. 2005; Foley and Faust 2010).

We also examined the influence of matriarchal social units on tusk circumference at lip and exposed tusk length, using a population of individually known elephants and their matriarchal social units. We tested the hypothesis that tusk size will vary between female elephants from different social units more than between members of the same social unit independently of stature or age. Female elephant social units mostly consist of genetically related females (Quellers genetic relatedness coefficient = 0.23; Archie et al. 2006; Wittemyer et al. 2009). Female social units therefore provide an approximate and inexpensive sampling unit for evaluating the influence of genetic relatedness on tusk similarity among individual elephants in the absence of pedigree data.

## Materials and Methods

### Spatial and temporal distribution of study populations

Data on contemporary elephant populations were collected between 2005 and 2013 from elephants captured and translocated by the Kenya Wildlife Service to reduce the negative interactions between elephants and humans (Omondi et al. 2004). Data on historical populations were collected between 1966 and 1968 from elephant culls. Original data sheets from the 1966 to 1968 elephant culls have been digitized and are available at: http://ufdc.ufl.edu/AA00013409/00007.

**Figure 1:**
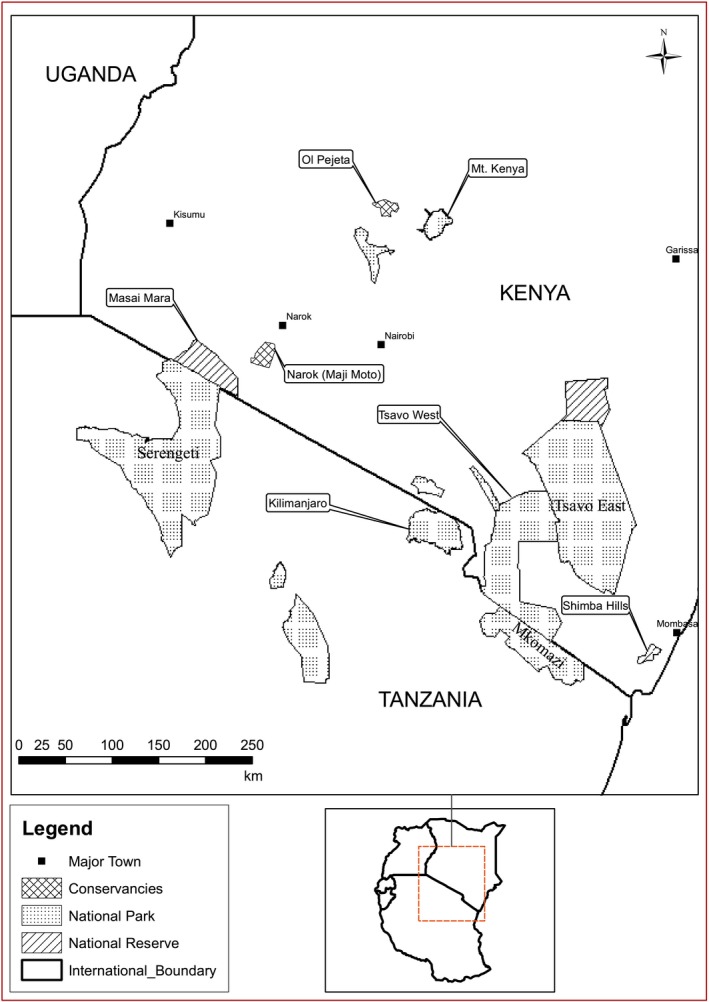
The location of elephant populations sampled in 1966–1968 (Tsavo East and Mkomazi National Parks) and in 2005–2013 (Shimba hills National reserve, Narok and Ol Pejeta wildlife conservancies and Tsavo East and Tsavo West National Parks).

For contemporary elephant populations (sampled between 2005 and 2013), we obtained data on age, sex, tusk sizes, and body measurements from elephants captured for translocation from a few locations in southern Kenya (Fig. 1). These translocations included the capture and movement of over 135 elephants from Shimba hills National Reserve to Tsavo East National Park in 2005, 9 elephants from Ol Pejeta Conservancy to Meru National Park in 2013, and 61 elephants from Siayapei Narok County to Masai Mara National Reserve in 2011 (Pinter‐Wollman et al. 2009; Mijele et al. 2011). In addition to data collected from live animals, we also measured aspects of morphology for 188 tusks recovered from dead animals in Tsavo National Park between 2005 and 2013 and 200 tusks confiscated from illegal trade in Kenya in 2013. For these tusks, elephant sex was determined from tusk size and morphology (Pilgram and Western 1986).

For historical elephant populations, we obtained data on age, sex, tusk, and body measurements for 200 elephants from Tsavo East National Park and 300 elephants from Mkomazi National Park culled in 1966 and 1968, respectively. These two National Parks are contiguous and form one ecosystem (Fig. 1).

### Body and tusk measurements and age estimation

Tusk circumference at the lip line, exposed tusk length (measured from the tusk tip to the lip line at the outer tusk curvature), shoulder height (measured from the sole of the foot to the crest of the scapula), and back length (measured from the occipital crest to base of the tail or anal flap) were measured from contemporary elephants. These measurements were taken while an elephant was recumbent following immobilization with a combination of etorphine hydrochloride (M99^®^ Norvatis South Africa) and hyaluronidase (Mijele et al. 2013). We also obtained measurements of tusk circumference at the base of the tusk and total tusk length from elephant tusks recovered in the Tsavo ecosystem between 2005 and 2013. For tusks confiscated from illegal ivory trafficking in Kenya in 2013, tusk circumference at the base of the tusk, total tusk length, exposed tusk length, and root length were also measured.

Identical measures to those recorded for contemporary elephants were extracted from the digital archives for elephants culled in Tsavo and Mkomazi between 1966 and 1968 (http://ufdc.ufl.edu/AA00013409/00007, accessed August 2014). These measurements included: tusk circumference at lip, tusk circumference at the base of the tusk, total tusk length, back length, and shoulder height (Laws et al. 1975). In all our analyses involving tusks, we used one tusk from each animal for consistency because most of the recent samples (2005–2013) consisted of data from a single tusk.

Ages of elephants sampled in 2005–2013 were visually estimated using morphological and developmental criteria described in Moss (1996) and Hanks (1972). In summary, relative shoulder height and tusk emergence is used to estimate ages of young animals up to 15–20 years and developmental changes associated with head morphology, body shape, and tusk size are used to estimates ages of older animals. Ages of elephants culled in 1966–1968 were estimated using the molar progression method. A detailed description of this method can be found in Laws (1966). The age estimates obtained from the visual and molar criteria described above are highly correlated, and they have been validated using known age individuals (Hanks 1972; Moss 1996; Lee et al. 2012). We use these age estimates for calculating variability in tusks measurements for animals of similar age and for estimating growth rate and asymptotic shoulder height in contemporary and historical elephant populations.

### Identification of individual animals and elephant families

Individual elephants and their family units were identified prior to translocation in order to aid the capture of entire family units in a single translocation event. This was carried out to minimize post‐translocation social stress caused by missing family members at release sites. Individual elephants were identified using features of the ear such as shape, slits, notches, holes, lumps, nicks, and vein patterns, in combination with body size and shape features for elephants above 10 years of age (Moss 1996). However, most calves below 10‐years‐old did not possess unique features for individual identification. These young calves were identified based on their size and association with known cows.

Elephant social groups are easily detectable because female elephants form stable social groups representing genetically related adult females and their offspring (Moss and Poole 1983; Archie et al. 2006). Adult males form less stable associations that are not entirely based on genetic relatedness (Moss and Poole 1983; Chiyo et al. 2011). In this study, we used matriarchal social units excluding all male animals as a proxy for family units. We defined a matriarchal social unit as the smallest group of adult female elephants and their offspring that were consistently found together during 1 year of monitoring.

### Statistical analyses

#### Variation in tusk size among individuals with similar age

Variation is the raw material upon which selection (anthropogenic or natural) can act upon. We examined existing variation in tusk size among individuals of similar age for ages in which we had measurements from five or more elephants. We calculated the coefficient of variation (CV) for several age groups separately for each sex among contemporary and historical elephant tusk samples. We used the CV as a measure of variability and tested whether there are significant differences in the coefficient of variation between males and females and between historical and contemporary elephant populations using Mann–Whitney U statistics.

#### Temporal variation in tusk size

**Figure 2:**
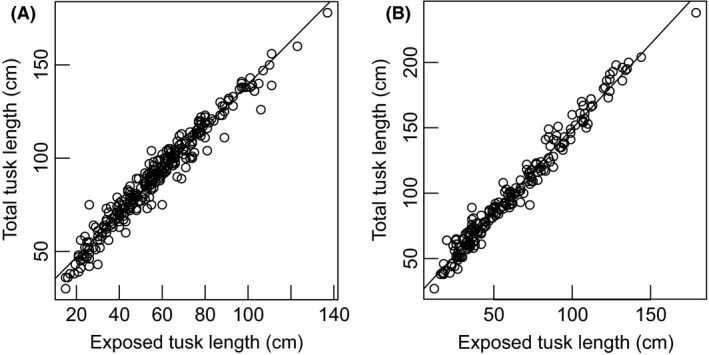
Predicting total tusk length from exposed tusk length in contemporary females (A, total tusk length = 23.73 + 1.159 × exposed tusk length*, R* ^2^_*adj*_ = 0.956), and males (B, total tusk length = 20.05 + 1.294 × exposed tusk length, *R* ^2^_*adj*_ = 0.974).

Measurements of total tusk length and tusk circumference at lip were compared between elephants taken from the Tsavo–Mkomazi ecosystem in 1966–1968 and those taken from elephant populations in southern Kenya in 2005–2013 (Fig. 1). Measurements of exposed tusk length were available for animals sampled in 2005–2013 whereas for culled elephants sampled in 1966 – 1968, we had measurements of total tusk length. To obtain comparable tusk measurements for these two time periods, we calculated total tusk length of the 2005–2013 data from predictions of a regression of exposed tusk length on total length obtained from recent measurements of confiscated ivory in Kenya in 2013 (Fig. 2).

Prior to statistical analysis, we divided our 2005–2013 data into two subsets. The first dataset consisted of survivors, that is, individuals born by 1970 or 35 years and older by 2005. This dataset was used to test for selection of large‐tusked individuals during the severe illegal harvesting for ivory of the late 1970s and early 1980s. The second dataset consisted of elephants born around 1995 and later. We used this data to test whether survivors of the severe poaching were able to transmit the “small tusk for stature or body size” trait to their offspring. For each dataset, sex, and dependent variable (i.e., tusk length and tusk circumference), we performed four analyses, using shoulder height and sampling period as single independent variables or in combination with or without an interaction term. We choose the most parsimonious of the four regression models conducted for each sex, dataset, and dependent variable using Bayesian Information Criteria, BIC (Tables S1 and S2). Shoulder height was used in the analyses above instead of age because the aging criteria in 2005–2013 and 1966–1968 were different. Shoulder height is tightly correlated with age in elephants; In Etosha National Park, Lindeque and Vanjaarsveld (1993) found *R* ^2^ between age and shoulder height of 0.95 and 0.94 in male and female elephants, respectively. The Amboseli and Addo National Parks data combined, produced *R* ^2^ between age and shoulder height of 0.93, and 0.91 in males and females, respectively (Shrader et al. 2006). Shoulder height and tusk size are likely to have correlated responses to ecological changes as both grow continuously throughout life in elephants, only slowing down later in life (Elder 1970; Hanks 1972). Using shoulder height *in lieu* of chronological age estimates minimized any effects due to temporal variation in ecological influences on tusk size in our model. In all regression analyses, including tusk length and tusk circumference as dependent variables, we centered shoulder height as a covariate so that our intercept would represent the differences in mean tusk length at mean of shoulder height.

#### Temporal differences in tusk allometry in the Tsavo–Mkomazi elephants

We also performed another four linear regression models for tusks collected only in the Tsavo–Mkomazi ecosystem between 2005–2013 and 1966–1968. In these analyses, we used tusk length as a dependent variable and tusk circumference at the base of the tusk and sampling period as single predictor variables or in combination including an interaction term. The best model was selected based on BIC (Table S3). Tusk circumference was centered in all statistical analyses. This was the only data taken from exactly the same location sampled in the 1960s. We used these analyses as a rough check for any spatial influences on results from analyses above, because we used tusk circumference instead of shoulder height (stature) as such data were not available from dead contemporary elephants. Tusk circumference is a good proxy for body size and age because it changed less across sampling periods in our analyses above. We expected tusk length at mean basal tusk circumference to be lower in contemporary elephants than elephants sampled in the 1960s.

#### Temporal differences in stature (shoulder height) and growth in elephant populations

Environmental changes over time can impact on growth rate and can have a profound influence on stature (Lee et al. 2013) and tusk size. To test whether small tusk size for body size we observed in contemporary elephants compared to elephants harvested in the 1966–1968 was driven by environmental influences, we examined differences in growth rate and asymptotic shoulder height between the two population samples. We used the von Bertalanffy growth model to determine growth rate and asymptotic shoulder heights in elephants sampled in 2005–2013 and 1966–1968. We predicted that if harsh environmental conditions were the drivers of small tusk size for body size we observed, then contemporary elephants should have a slower growth rate and a smaller asymptotic shoulder height. We use the likelihood ratio tests of Kimura (1980) to evaluate differences in the growth parameter (*K)* and the asymptotic growth parameter (*L∞*). These parameters were calculated using fishmethods (Nelson 2014), a package of the R statistical software (R Development Core Team 2015).

#### Variation in tusk size between elephant social groups

To test whether variation in exposed tusk length and tusk circumference at the lip line is larger between elephant social groups than within elephant social groups, we used analysis of (co)variance. Prior to statistical analyses, we transformed values of exposed tusk length, tusk circumference at lip, and elephant shoulder height in to their natural logarithms in order to linearize the relationships between shoulder height and tusk size measurements. We selected seven elephant families consisting of at least five or more individuals with tusk measurements. Exposed tusk length and tusk circumference at the lip line were used as dependent variables in separate analyses. Shoulder height and family ID were used as independent variables either singly or in combination with or without an interaction term. The results of the best model selected based on Bayesian Information Criteria are reported. To minimize the potential influence of variation in illegal harvesting across social groups, we used only individuals born after 1990 in a second set of analyses. We performed analyses of (co)variance as above for the only three social groups with at least three or more born after 1990.

All statistical analyses were performed using the R software for statistical computing (R Development Core Team 2015).

## Results

### Variation in tusk sizes among elephants of similar age and sex

Within animals of a given age, tusk length was quite variable for historical elephant samples collected in the 1966–1988 and contemporary elephants sampled in 2005–2013 as indicated by the mean CV across age groups (Table 1). Female and male elephants of similar age sampled in 1966–1968 had a similar CV (median of 11.1 for females and 11.5 for males, *U* = 227, *P* = 0.518). In contemporary elephants (sampled in 2005–2013), the CV for tusk length among elephants of similar age was large, indicating that tusk length was more variable among the sexes compared with elephant samples collected in 1966–1968. The median CV was 14.5 in females and 21.4 in males and was statistically different (*U *=* *1.0, *P* = 0.007). The median CV of tusk length in females sampled between 2005 and 2013 was significantly higher than that of females sampled in 1966–1968 (*U *=* *50, *P* = 0.024). Similarly, the median CV of tusk length among males sampled in 2005–2013 was significantly higher than that for males sampled in 1966–1968 (*U *=* *0, *P* > 0.001). Tusk diameter at lip was also variable between elephants sampled in 2005–2013 and 1966–1968 and between females and males (Table 1). In female elephants sampled in 1966–1968, the median CV for tusk circumference was 8 and was not significantly different from that of males which was 9.1 (*U *=* *169, *P* = 0.107). Females sampled in 1966–1968 had a median CV of 8 which was significantly lower than that for females sampled in 2005–2013 which was 14 (*U *=* *35, *P* = 007). In contemporary males (sampled in 2005–2013), the median CV for tusk diameter at the lip was 22.1 and was not significantly different from that of males sampled in 1966–1968 which was 9.1 (*U *=* *42, *P* = 0.356).

**Table 1:** The statistics for the coefficient of variation for tusk length and tusk circumference at the lip across age groups for male and female elephants sampled in 1966–1968 and 2005–2013

Statistic	1966–1968		2005–2013	
	Female	Male	Female	Male
Tusk length				
Mean	10.5	11.3	14.5	21.4
Median	11.1	11.5	14.3	23.3
SD	3.1	2.9	2.9	6.1
Range (min.–max.)	5.3–18.4	6.6–17.0	8.8–18.3	17.5–33.0
Number of age groups	27	19	8	5
Mean number of animals per age group	7.4	9	7.1	7.2
Tusk circumference at the lip				
Mean	8.5	9.6	13.9	16.3
Median	8	9.1	14	22.1
SD	2.7	2.4	4.2	9.2
Range (min.–max.)	2.6–14.2	5.5–13.6	6.9–19.2	7.8–27.2
Number of age groups	25	19	8	6
Mean number of animals per age group	7.6	9.4	7.1	7.7

### Temporal variation in tusk size for stature: the effects of illegal harvesting

**Figure 3:**
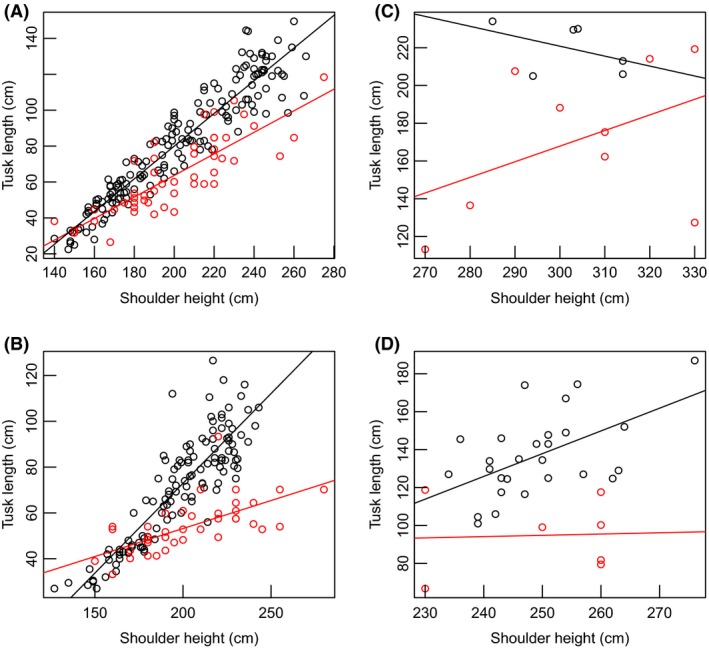
Variation in tusk length as a function of shoulder height for the elephant samples collected in 1966 – 1968 (black) and 2005–2013 (red). Contemporary males (A) and females (B) born after 1995 and onwards and males (C), and females (D) born by 1970 (survivors) compared with male and female elephants of similar age sampled in 1966–1968. Lines of model fit are for visualization only.

Mean tusk length at average stature (shoulder height) among survivors (elephants sampled in 2005–2013 that were born by 1970) of the illegal killing for ivory in the 1970s/1980s declined by 22% in male elephants, while in female elephants, it declined by 37% compared to elephants of similar size sampled in 1966–1968 (Table 2, Fig. 3C & 3D). The rate of tusk growth relative to stature among survivors was not significantly different from the 1966–1968 average as a baseline for both females and males because our best models for each sex did not include an interaction between shoulder height and sampling period (Table S1). The lack of a difference in growth rate is to be expected as survivors were older animals, whose growth rate is usually dramatically low. Such low rates would require large sample sizes to detect an effect.

**Table 2:** Parameters from models predicting change in mean tusk length in African elephants between 1966–1968 (historical elephant population) and the 2005–2013 (contemporary elephants). Tusk measurements are in centimeters

Regression covariates	Coefficient	Standard error	*T* statistic	Probability
Contemporary elephants born in 1995 onwards compared with 1966–1968 elephants of similar age				
Males				
Intercept	81.462	0.849	95.940	<0.0001
Shoulder height	28.899	0.825	35.030	<0.0001
2005–2013 relative to 1966–1968	−16.831	1.750	−9.620	<0.0001
Shoulder height × 2005–2013	−9.843	1.911	−5.150	<0.0001
Females				
Intercept	72.515	0.981	73.907	<0.0001
Shoulder height	21.471	1.030	20.841	<0.0001
2005–2013 relative to 1966–1968	−19.453	1.966	−9.897	<0.0001
Shoulder height × 2005–2013	−14.778	1.838	−8.039	<0.0001
Contemporary elephants born by 1970 (Survivors) compared with 1966–1968 elephants of similar age				
Males				
Intercept	219.580	13.010	16.880	<0.0001
2005–2013 relative to 1966–1968	−48.040	16.800	−2.860	0.0134
Females				
Intercept	136.827	3.756	36.424	<0.0001
Shoulder height	8.628	3.400	2.538	0.0164
2005–2013 relative to 1966–1968	−42.686	8.284	−5.153	<0.0001

For animals born in 1995 and later, mean tusk length for average stature declined by 21% in males and by 27% in females relative to the 1966–1968 average as baseline (Table 2). There was also a decline in the rate of tusk growth with respect to stature in males and females born after the severe poaching of the 1970s and the 1980s (Table 2, Fig. 3A & 3B).

Tusk circumference at lip for an average stature in male elephants that survived the illegal killing for ivory declined by 8% relative to the 1966–1968 average. In female elephants, tusk circumference declined by 11% between 2005–2013 and 1966–1968 (Table 3). However, tusk circumference at lip in male elephants born after 1995 and elephants of similar ages from the 1966–1968, declined by 5%. For females, however, the best model did not include sampling period suggesting tusk circumference was not significantly different between 2005–2013 and 1966–1968 (Table S2). The rate of growth in tusk circumference relative to growth in stature was not significantly different between 2005–2013 and 1966–1968 for either elephant survivors or their offspring as our best models did not have an interaction between shoulder height and sampling period (Table S2). The lack of a statistically significant difference in growth rates of tusk circumference between male elephants sampled in 2005–2013 and animals of similar age from 1966 to 1968 suggests a lack of statistical power to detect small differences in growth.

**Table 3:** Parameters from models predicting change in mean tusk circumference at lip in African elephants between 1966–1968 (historical elephant populations) and 2005–2013 (contemporary elephant populations). Tusk measurements are in centimeters

Regression covariates	Coefficient	Standard error	*T* statistic	Probability
Contemporary elephants born in 1995 onwards compared with 1966–1968 elephants of similar age				
Males				
Intercept	20.062	0.185	108.209	<0.0001
Shoulder height	4.491	0.162	27.771	<0.0001
2005–2013 relative to 1966–1968	−1.120	0.376	−2.977	0.0033
Females				
Intercept	15.248	0.138	110.200	<0.0001
Shoulder height	2.466	0.139	17.760	<0.0001
Contemporary elephants born by 1970 (Survivors) compared with 1966–1968 elephants of similar age				
Males				
Intercept	44.708	1.049	42.634	<0.0001
2005–2013 relative to 1966–1968	−3.565	1.253	−2.845	0.0108
Females				
Intercept	24.755	0.374	66.112	<0.0001
Shoulder height	1.148	0.416	2.761	0.0089
2005–2013 relative to 1966–1968	−2.703	0.909	−2.973	0.0052
Shoulder height × 2005–2013	−1.559	0.748	−2.085	0.0440

### Temporal changes in tusk allometry in the Tsavo–Mkomazi elephants

**Figure 4:**
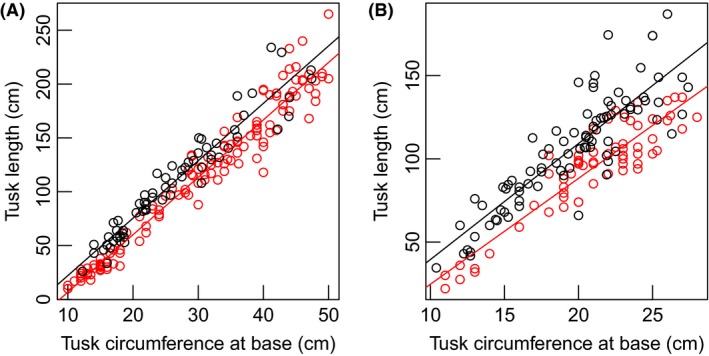
Variation in tusk length as a function of tusk circumference at base in (A) male and (B) female African elephants from Tsavo National Park in 1966–1968 and 2005–2013. The black circles show data for the 1966–1968 tusk samples, and the red or gray circles show the 2005–2013 tusk samples.

Mean tusk length at average basal tusk circumference was lower in contemporary Tsavo elephants compared to Tsavo elephants in 1966–1968 (Table 4, Fig. 4). The most parsimonious covariate models (Table S3) indicated that tusk length in male elephants was lower by 12.4% in 2005–2013 than in 1966–1968 (Table 4, Fig. 4A). During the same period, tusk length declined by 21.2% in females (Table 4, Fig 4B).

**Table 4:** Change in mean tusk length predicted by tusk circumference at the base for male and female elephants from the Tsavo ecosystem between 1966–1968 and 2005–2013. Tusk measurements are in centimeters

Regression covariate	Coefficient	Standard error	*T* statistic	Probability
Males				
Intercept	123.763	1.807	68.474	<0.0001
Circumference	60.073	1.065	56.385	<0.0001
2005–2013 relative to 1966–1968	−15.382	2.239	−6.868	<0.0001
Females				
Intercept	111.362	1.529	72.850	<0.0001
Circumference	28.869	1.165	24.780	<0.0001
2005–2013 relative to 1966–1968	−23.569	2.341	−10.070	<0.0001

### Differences in elephant stature and growth for the 1966–1968 and 2005–2013 samples

The Von Bertalanffy growth parameters for male and female elephants sampled in 2005–2013 and 1966–1968 were statistically different, but the difference was small (females *χ* ^2^ = 71.59, df = 3, *P* < 0.001 Males: *χ* ^2^ = 25.91, df = 3, *P* < 0.001). More specifically, female and male elephants sampled in 2005–2013 had slightly higher asymptotic shoulder heights compared to elephants sampled in 1966–1968 (Table 5). The growth rate was also slightly higher in contemporary male and female elephants, but the difference was not statistically significant for males (Table 5).

**Table 5:** Differences in Von Bertalanffy growth parameters and standard errors for shoulder height between contemporary elephants sampled in 2005–2013 and historical populations sampled in 1966–1968 from southern Kenya. Shoulder height measurements are in centimeters

	2005–2013	1966–1968	*P* values
Females			
Asymptotic shoulder height (*L*∞)	253.03 ± 5.64	245.48 ± 2.12	0.002
Growth rate (*K*)	0.170 ± 0.035	0.129 ± 0.008	0.002
Shoulder height at time 0 (*t*_0_)	−2.69 ± 1.15	−4.3 ± 0.41	0.003
Sample size (*N*)	110	321	
Males			
Asymptotic shoulder height (*L*∞)	313.11 ± 14.19	296.17 ± 6.62	0.013
Growth rate (*K*)	0.075 ± 0.018	0.084 ± 0.007	0.317
Shoulder height at time 0 (*t*_0_)	−6.46 ± 2.11	−5.59 ± 0.52	0.301
Sample size (*N*)	89	282	

### Variation in tusk size between elephant families

The mean log of tusk length and the change in tusk length with stature (shoulder height) differed between elephant social units from Shimba hills (social unit: *F*_6_ = 2.777, *P* = 0.025; shoulder height × social unit, *F*_6_ = 4.935, *P* = 0.001). Tusk length accounting for stature varied among social groups even when we restricted our analyses to only individuals born after 1990 (social unit: *F*_2_ = 4.547, *P* = 0.039).

Tusk circumference was marginally more variable across elephant social groups than within elephant social groups (social unit: *F*_6_ = 2.208, *P* = 0.061). This pattern of variation persisted even when we restricted our analyses to animals born after 1990 (social unit: *F*_2_ = 9.161, *P* = 0.011; shoulder height × social unit: *F*_2_ = 2.305, *P* = 0.170).

## Discussion

Tusk size in African elephants experienced a substantial decline in 2005–2013 relative to the 1960s; a period covering two elephant generations. The decline in tusk size supports a hypothesis of selective poaching of animals with large tusks in the 1970s/1980s. Although our sample of contemporary elephants came from a much larger area than the 1966–1968 samples, an analysis of tusks collected from the same location during the 1966–1988 and 2005–2013 time periods indicated a decline in tusk length at mean tusk circumference. The robustness of our result to spatial variation in sampling during the 1960s and 2000s is consistent with the spatial genetic structure of contemporary populations which shows that the majority of our samples came from populations of elephants sharing a single mitochondrial haplotype cluster (Okello et al. 2008). The mitochondrial locus is maternally inherited, and the mitochondrial haplotype distribution varies spatially in elephants due to strong female philopatry (Okello et al. 2008). The decline in tusk size is congruent with studies in temperate regions showing that harvesting of wild ungulate populations by humans can lead to directional selection against traits that are sought after by hunters such as large horns (Coltman et al. 2003; Garel et al. 2007; Festa‐Bianchet et al. 2014).

Our contemporary elephant samples came from animals involved in conflict with humans suggesting that contemporary elephants were located in habitats highly fragmented or degraded by human activities. Elephant populations isolated by fragmentation can become locally overabundant causing elevated elephant densities (van Aarde and Jackson 2007). These factors can lead to nutritional stress or forage limitation, which could cause a reduction in tusk size we observed in this study. A similar reduction in hunter preferred traits as a result of environmental and population density‐dependent effects have been documented elsewhere for other species (Garel et al. 2007; Hedrick 2011). On the contrary, most habitats occupied by contemporary elephants in our study have been experiencing bush encroachment due to relatively low elephant densities compared to the 1960s (Leuthold 1996), indicating that there is no forage limitation facing contemporary elephants. In support, asymptotic shoulder height was slightly higher for the contemporary elephant samples compared to the historical elephant samples, suggesting that nutritional stress was not an important factor influencing the reduction in tusk size we observed in our study.

It is also conceivable that a rapid increase in stature and slow growth in tusk due to density‐dependent forage or nutritional effects could lead to a reduction in tusk size for stature because mammalian teeth are influenced to a lesser extent by environmental factors than body stature (Cardoso 2007; Conceição and Cardoso 2011). The differential response of growth in stature and tusk size to environmental variation is unlikely to have caused a reduction in tusk size for stature for two reasons. Firstly, unlike typical mammalian teeth, tusks in elephants grow almost throughout life (Laws 1966; Elder 1970) like skeletal structures that determine stature such as shoulder height and are more likely to be sensitive to environmental and nutritional influences (Lee et al. 2013). Secondly, we also observed a large reduction in tusk length for mean tusk diameter in contemporary elephants compared to historical elephant populations from the Tsavo–Nkomazi elephants. This result suggests that the reduction in tusk size for shoulder height is not merely caused by differential growth in tusk size and shoulder height in response to ecological variation.

The reduction in tusk size we observed was large in females compared to males. Females occur in large groups whereas males are more solitary than females but may occur in association with other males in small all‐male groups or in temporary association with large groups comprising of family herds (Moss and Poole 1983; Poole and Moss 1989; Chiyo et al. 2011). Selection of animals with relatively large tusks is more likely for elephants in large groups typical of elephant family groups or mixed groups, than for solitary individuals typical of males. Selection of a single animal with large tusks within an elephant group increases the per capita monetary returns while minimizing the risk of detection when extracting tusks from an entire herd in a single incident. An alternative explanation for sex differences in the reduction of tusk length for age could also be genetically driven as seen in the expression of tusklessness (Jachmann et al. 1995; Whitehouse 2002). The expression of tusklessness involves sex‐influenced penetrance and expressivity (Jachmann et al. 1995). The prevalence of tusklessness is usually higher in female than in male elephants, and this phenomenon appears to be exacerbated by poaching (Jachmann et al. 1995; Whitehouse 2002). A similar phenotype in humans called maxillary lateral incisor hypodontia shows an identical pattern of sex‐influenced expression (Alvesalo and Portin 1969; Pinho et al. 2009). The similarities in the expression of tusklessness and maxillary lateral incisor hypodontia suggest a conserved mammalian pattern of incisor inheritance and phenotype expression.

We also found greater differences in elephant tusk size (i.e., tusk length and circumference) between social groups than within social groups. The influence of social group on tusk size in a subsample of animals within social groups born after 1990 also showed greater variation in tusk size between than within elephant social groups. This latter analysis suggests that differential harvesting among elephant social groups was not the major driver of variation in tusk size among social groups. Instead, the similarity in tusk size within social groups suggests the influence of genetics on tusk size in elephants. This is because a female elephant belonging to a social group is usually genetically related to others in the same social group more than to any random member in the population. For example, Queller's genetic relatedness coefficient for core social units is known to vary from 0.15 to 0.234 (Archie et al. 2006; Gobush et al. 2008; Wittemyer et al. 2009). If tusk length and tusk circumference are heritable, we should therefore find greater similarity within social groups than between social groups as observed in this study. Although our evidence for the role of genetics on tusk size is indirect, studies on mice, baboons (Hlusko et al. 2011), and humans (Alvesalo and Tigerstedt 1974) have established that incisor size, an homologous tooth to a tusk in elephants is an heritable trait with substantial genetic influence (Koussoulakou et al. 2009). For example, maxillary incisor heritability (*h* ^2^) is 0.37 in mice, 0.49 in baboons, and 0.57 in humans. These studies indicate that incisor tooth sizes are highly heritable in mammals. Our result should be treated as preliminary until a definitive study on heritability of tusk size in elephants using data from long‐term studies of populations where individuals and their parentage are known is carried out.

## Conclusions

This study provides empirical evidence for selection of elephants with large tusk size for age and suggests that illegal ivory harvesting is a major driver of reduction in tusk size for age in African elephants. The study contributes to our understanding of the increasing role humans play in phenotypic evolution of wild populations. We suggest long‐term monitoring of traits targeted by hunters in harvested populations of wild free ranging mega‐herbivores to determine the negative impact of harvesting and identify populations potentially at risk from compromised adaptive potential.

## Conflict of Interest

None declared.

## Supporting information


**Table S1**. A comparison of models predicting tusk length in African elephants using Bayesian Information Criteria.
**Table S2.** A comparison of models predicting tusk circumference in African elephants using Bayesian information criteria.
**Table S3.** A comparison of models predicting tusk length in Tsavo National Park from tusk circumference and sampling period (1966–1968 and 2005–2013) using a Bayesian information criteria.
