# Supplementary material for: Illegal tusk harvest and the decline of tusk size in the African elephant
Source: Ecol Evol. 2015 Oct 22;5(22):5216–29. doi: 10.1002/ece3.1769 (PMC6102531; doi:10.1002/ece3.1769)
Supplement: Supplementary file 1 — Table S1. A comparison of models predicting tusk length in African elephants using Bayesian Information Criteria. Table S2. A comparison of models predicting tusk circumference in African elephants using Bayesian information criteria. Table S3. A comparison of models predicting tusk length in Tsavo National Park from tusk circumference and sampling period (1966–1968 and 2005–2013) using a Bayesian information criteria. [file ECE3-5-5216-s001.doc]

**Supporting Information**

**Table S1**: A comparison of models predicting tusk length in African elephants using Bayesian Information Criteria. Period in the model covariates represents the 1966-68 and 2005-13 sampling periods as categorical variables. Elephants sampled in 2005-13 are divided into those born around 1995 and later and those born by 1970s compared with similar aged animals sampled in 1966-68.

| **Model** | **BIC** | **ΔBIC** |
| --- | --- | --- |
| **Elephants born in 1995 and onwards** |  |  |
| ***Tusk length in males*** |  |  |
| Shoulder height + Period + (Shoulder height x Period) | 1633.56 | 0.00 |
| Shoulder height + Period + Shoulder height | 1653.65 | 20.09 |
| Shoulder height | 1716.85 | 83.29 |
| Period | 2049.65 | 416.09 |
| ***Tusk length in females*** |  |  |
| Shoulder height + Period + (Shoulder height x Period) | 1225.98 | 0.00 |
| Shoulder height + Period + Shoulder height | 1276.31 | 50.34 |
| Shoulder height | 1336.20 | 110.22 |
| Period | 1433.33 | 207.35 |
| **Elephants born by 1970** |  |  |
| ***Tusk length in males*** |  |  |
| Shoulder height + Period + (Shoulder height x Period) | 154.44 | 2.05 |
| Shoulder height + Period + Shoulder height | 153.10 | 0.70 |
| Shoulder height | 158.84 | 6.44 |
| Period | 152.40 | 0.00 |
| ***Tusk length in females*** |  |  |
| Shoulder height + Period + (Shoulder height x Period) | 309.93 | 0.43 |
| Shoulder height + Period + Shoulder height | 309.50 | 0.00 |
| Shoulder height | 327.01 | 17.51 |
| Period | 312.39 | 2.89 |

**Table S2:** A comparison of models predicting tusk circumference in African elephants using Bayesian information criteria. Period in the model covariates represents the 1966-68 and 2005-13 sampling periods as categorical variables. Elephants sampled in 2005-13 are divided into those born around 1995 and later and those born by 1970s compared with similar aged animals sampled in 1966-68.

| **Model** | **BIC** | **ΔBIC** |
| --- | --- | --- |
| **Elephants born in 1995 and onwards** |  |  |
| ***Tusk circumference in males*** |  |  |
| Shoulder height + Period + (Shoulder height x Period) | 998.38 | 5.20 |
| Shoulder height + Period + Shoulder height | 993.18 | 0.00 |
| Shoulder height | 996.62 | 3.44 |
| Period | 1316.94 | 323.76 |
| ***Tusk circumference in females*** |  |  |
| Shoulder height + Period + (Shoulder height x Period) | 648.86 | 7.44 |
| Shoulder height + Period + Shoulder height | 644.44 | 3.02 |
| Shoulder height | 641.42 | 0.00 |
| Period | 816.62 | 175.20 |
| **Elephants born by 1970 or before** |  |  |
| ***Tusk circumference in males*** |  |  |
| Shoulder height + Period+(Shoulder height x Period) | 106.80 | 5.43 |
| Shoulder height + Period + Shoulder height | 103.94 | 2.57 |
| Shoulder height | 108.80 | 7.42 |
| Period | 101.37 | 0.00 |
| ***Tusk circumference in females*** |  |  |
| Shoulder height + Period + (Shoulder height x Period) | 194.71 | 0.00 |
| Shoulder height + Period + Shoulder height | 195.55 | 0.84 |
| Shoulder height | 200.48 | 5.77 |
| Period | 195.36 | 0.65 |

**Table S3:** A comparison of models predicting tusk length in Tsavo National Park from tusk circumference and sampling period (1966-68 and 2005-13) using a Bayesian information criteria.

| **Model covariates** | **BIC** | **ΔBIC** |
| --- | --- | --- |
| **Males** |  |  |
| Circumference + Period + (Circumference x Period) | 1549.35 | 5.23 |
| Circumference + Period | 1544.12 | 0.00 |
| Circumference | 1581.57 | 37.45 |
| Period | 2082.26 | 538.14 |
| **Females** |  |  |
| Circumference + Period + (Circumference x Period) | 1178.23 | 0.63 |
| Circumference + Period | 1177.61 | 0.00 |
| Circumference | 1250.66 | 73.05 |
| Period | 1427.04 | 249.44 |
